# Wheat Drought Management: A Broader Prospect

**DOI:** 10.3390/plants15172653

**Published:** 2026-08-29

**Authors:** Asfa Batool, Shi-Sheng Li, Wei Tu, Yun-Li Xiao, Ting Zhou, Hongyuan Du

**Affiliations:** 1 Key Laboratories of Economic Forest Germplasm Improvement and Comprehensive Resources Utilization of Hubei Province, Collaborative Innovation Center for the Characteristic Resources Exploitation of Dabie Mountains, College of Biology and Agricultural Resources, Huanggang Normal University, Huanggang 438000, China; shishengli@hgnu.edu.cn (S.-S.L.); tuwei@hgnu.edu.cn (W.T.); xiaoyunli0817@126.com (Y.-L.X.); 2 School of Agriculture and Biomanufacturing, Zhengzhou University, Zhengzhou 450001, China; zhoutt@zzu.edu.cn

**Keywords:** food security, trichomes, superoxide dismutase, ascorbate peroxidase, genetic engineering, abscisic acid (ABA)

## Abstract

Wheat (*Triticum aestivum* L.) is considered one of the most important cereals globally, contributing significantly to the human population’s caloric and protein requirements. Therefore, ensuring a sufficient yield of wheat for global consumption plays a significant role in maintaining food security in different parts of the world. With increasing demand and dwindling production capacity, due to increasingly uncertain growing conditions, projections indicate that there should be an upswing of 60–70% in wheat productivity by 2050 to fulfill the requirement. However, drought represents the most significant and widespread abiotic limitation to global wheat production, currently resulting in approximately 10% yield losses worldwide. Furthermore, each additional 1 °C increase in temperature is anticipated to decrease staple calorie production by 4.4%. The factors contributing to drought in wheat, as well as its impact on the plant’s biochemical, physiological, and morphological structures, include altered rainfall patterns, elevated atmospheric CO_2_ levels, increased temperatures, hot and dry winds, and restricted soil water availability. These factors initiate a series of morphological, physiological, and biochemical disruptions that hinder wheat growth and productivity. Drought impact on wheat starts at biochemical levels through reactive oxygen species (ROS) generation and degradation of chlorophylls, and tolerance to stress is influenced by a polygenic system where numerous genes contribute minor effects and interact significantly with environmental factors transitioning to osmoprotectants. At the physiological level, drought alters the water content in the plant body, leading to reduced net photosynthetic rates, stomatal conductance, transpiration rates, and water utilization efficiency. At the morphological level, drought impacts all kinds of structures such as roots, shoots, leaves and reproductive parts. To counter these effects, wheat develops a set of tolerant mechanisms called drought escape, avoidance and tolerance. An increase in trichomes and leaf waxes, alteration of root–shoot ratios, the staying green phenomenon, production of stress proteins like proline, activity of enzymes including superoxide dismutase (SOD), ascorbate peroxidase, catalase, etc., osmotic adjustment, abscisic acid (ABA) accumulation, expression of dehydration proteins called dehydrin, etc., contribute towards drought tolerance. This comprehensive review investigates the intricate interactions between drought and various wheat genotypes, emphasizing their substantial impacts on plant physiology, biochemistry, growth dynamics, and grain yield. Additionally, this review assesses a variety of genetic and biotechnological strategies aimed at enhancing the resilience of wheat genotypes to drought stress. By integrating recent research findings with practical applications, this review provides a detailed framework for improving the adaptive capacity of wheat plants to withstand the escalating threats of drought stress, thereby supporting sustainable wheat production in a changing climate. Addressing drought stress through genetic and biotechnological management practices is crucial for maintaining wheat productivity.

## 1. Introduction

Wheat is one of the world’s most important cereal crops because it contributes substantially to human calorie and protein supply and underpins food security in many regions [1,2,3]. Demand pressures are rising at the same time that production environments are becoming more unstable, with estimates that wheat output must increase by about 60–70% by 2050 to meet future needs [2,4,5]. Drought is now recognized as one of the most serious abiotic threats to wheat production worldwide, and its importance is increasing with climate-driven shifts in rainfall and temperature [6,7,8]. This threat is especially acute in rain-fed, arid, and semi-arid production systems, where water scarcity directly constrains crop establishment, biomass accumulation, grain set, and yield stability [2,9,10]. Newer global assessments further suggest that the area exposed to drought in major wheat-growing regions is likely to expand substantially during this century, reinforcing the need for forward-looking management [3]. Drought affects wheat at multiple biological levels, from whole-plant morphology to cellular metabolism and gene regulation [6,11,12]. Common consequences include reduced relative water content, stomatal closure, lower chlorophyll retention, impaired photosynthesis, disrupted carbon fixation, oxidative damage, earlier senescence, and poorer grain filling [8,13,14]. Meta-analytic studies done on 48 previously published articles indicate that drought can reduce wheat grain yield by 57.32% on average and grain protein yield by 46.04%, even while grain protein concentration tends to rise as yield declines [15]. The effect of drought is not uniform because yield loss depends strongly on the timing, intensity, and duration of stress [8,16]. Flowering and grain filling are consistently identified as highly sensitive stages, although tillering and anthesis-related transitions also contribute importantly to final losses under water deficit [8,16,17]. Continuous drought appears more damaging than terminal drought alone [15]. In one cross-environment meta-analysis, continuous drought reduced yield by 83.60%, compared with 26.43% under terminal drought [15]. Drought also rarely acts in isolation in farmers’ fields, and combined heat plus drought during grain formation further disrupts meiosis, pollen function, ovule survival, endosperm development, and final grain mass [18]. Wheat does possess endogenous defense strategies, including escape, avoidance, tolerance, and recovery responses [6,14,17]. These responses involve osmotic adjustment, proline accumulation, changes in antioxidant enzyme activity, abscisic acid (ABA) signaling, altered root–shoot balance, stay-green behavior, and membrane stabilization [6,13,14]. Still, intrinsic tolerance is often insufficient under severe, prolonged, or compound stress, which is why recent reviews increasingly argue for integrated management rather than single-trait solutions [8,19]. The strongest long-term pillar of wheat drought management remains genetic improvement because cultivars with greater drought tolerance and water-use efficiency can reduce risk without requiring costly inputs [2,5,9]. However, breeding progress is inherently difficult because drought tolerance is polygenic, strongly environment-dependent, and hard to consistently phenotype across target regions [1,7,13]. Recent literature, therefore, emphasizes a synergistic breeding model that combines conventional selection with physiological trait-based screening, marker-assisted approaches, genomic tools, and, increasingly, gene editing [5,13,20]. Genetic gains also depend on expanding the usable diversity of wheat through landraces, synthetics, wild relatives, and introgression populations [2,5,12]. New phenotyping and multi-omics approach now make it easier to identify useful genomic regions, physiological markers, and metabolic signatures associated with drought adaptation [1,3,10]. Several recent reviews also highlight accessible screening tools, including carbon isotope discrimination, canopy temperature, stress indices, and early-stage morpho-physiological traits, as practical bridges between gene discovery and field selection [4,10,11]. Agronomic adaptation is the second major pillar because management can reduce crop exposure to drought even when genetic tolerance is incomplete [9,11,17]. Commonly proposed measures include early sowing, conservation tillage, mulching, intercropping, crop and variety choice, micro-irrigation, seed priming, and water budgeting [13,21]. Post-drought recovery also matters: winter wheat often recovers substantially after rewatering, especially when stress is mild or short, although recovery varies by growth stage and production environment [22]. Recent work broadens management further by incorporating biological and material-based interventions that improve soil–water relations or plant stress buffering [19]. Reviews now point to plant-growth-promoting bacteria, microbial inoculants, biochar, nanoparticles, and other biostimulant-type inputs as promising complements to breeding and agronomy rather than replacements for them [23]. The common theme across these newer approaches is not that one technology solves drought but that combinations of soil management, crop genetics, and stress physiology are more likely to produce durable field resilience [2,5,19]. In sum, wheat drought management is best introduced as a systems challenge rather than a single stress problem. The newer literature supports a broader prospect in which drought-resilient cultivars, adaptive agronomy and emerging biological or molecular tools are integrated to sustain wheat productivity under increasingly erratic climates [3,11,18]. Consequently, the primary aim of this research is to examine drought stress, including its causes, impacts, tolerance, and management in wheat, which assists plant breeders in identifying diverse strategies to mitigate or alleviate the effects of drought stress in wheat crops, thereby ensuring global food security.

### Methodological Framework

This content synthesizes information from various secondary sources, including peer-reviewed research and review articles published in reputable journals. It integrates insights and data from a diverse array of references accessed through multiple platforms and resources that have been examined.

## 2. Results

### 2.1. Drought on Wheat: Causes

Drought is the most severe and widespread abiotic constraint limiting global wheat production, already causing roughly 10% yield losses worldwide, with every additional 1 °C of warming projected to reduce staple calorie production by 4.4% [10,24]. The impact of drought on wheat and its effects on the biochemistry, physiology, and morphological structure of the wheat plant span changing rainfall patterns, elevated atmospheric CO_2_, increased temperatures, hot and dry winds, and limited soil water availability. These drivers trigger a cascade of morphological, physiological, and biochemical abnormalities that retard wheat growth and productivity [6,25]. Drought stress in wheat arises from a combination of climatic, edaphic, and atmospheric factors that reduce water availability to the crop. Changing rainfall patterns, increased atmospheric CO_2_ levels, rises in atmospheric temperature, and hot and dry winds are the major causes of drought stress [6]. The phenomenon of drought stress in plants is characterized by its intricate nature, arising from many environmental factors, including limited soil water availability, elevated soil salinity levels, and increased ambient temperature. The latter is referred to as physiological drought, where even if water is present in the soil, plants cannot absorb it due to high osmotic pressure or low temperatures [25]. A rising problem of global warming is predicted to amplify the frequency and severity of drought in the near future, bringing a lack of water resources that influences morphological, biochemical, physiological, and molecular attributes of the plants [26]. Under the influence of monsoon climates, precipitation is generally low during the growing season of wheat in some regions, and the asynchrony between the rainy season and the critical water-demanding period of wheat leads to frequent drought stress [22]. Drought stress frequently intersects with other stressors, such as elevated temperatures, exacerbating the overall impact on wheat yield. A recent study found that a mere 40% loss in soil moisture might lead to a significant decrease of approximately 21% in wheat yield [25]. Drought-stressed loss in wheat yield likely exceeds losses from all other causes, since both the severity and duration of the stress are critical [26]. Globally, drought can reduce crop yield and productivity by up to 70% [27,28].

### 2.2. Drought Effects on Wheat

#### 2.2.1. The Physiological Effects of Drought on Wheat

Drought stress severely restricts winter wheat growth and development by altering its water content, which directly reduces net photosynthetic rate, stomatal conductance, transpiration, and water use efficiency [22,29,30]. The physiological effects of drought vary significantly depending on the intensity and duration of the water deficit (Table 1), the plant’s developmental stage, and the specific genotype’s adaptive capacity [22,29,31,32]. Drought stress significantly reduces leaf relative water content, water potential, osmotic potential, and turgor pressure, fundamentally disrupting the plant’s hydration status [29,33,34]. This loss of turgor pressure directly retards cell extensibility, embryo development, and overall plant growth [6]. Stomatal closure is a primary physiological response to drought that limits carbon dioxide uptake and immediately reduces the photosynthetic rate [35]. Drought significantly decreases stomatal conductance and transpiration rates across diverse wheat genotypes [29,30,36]. This stomatal restriction is a drought-avoidance strategy, though it creates a CO_2_ shortage that provokes photorespiration and lowers overall photosynthetic output [29,37]. Drought adversely affects photosystem II by slowing electron transport, increasing non-photochemical quenching, and ultimately lowering the relative moisture content in the cell [6,38]. Severe drought stress significantly reduces the photosynthetic capacity of crops, causing a decrease in chlorophyll content, net saturated photosynthetic rate, and the maximum quantum yield of photosystem II (Fv/Fm) [38,39,40]. Drought-induced oxidative stress further damages cellular structures, including chloroplasts, which impairs photosynthetic productivity. Drought negatively influences nutrient assimilation and mobilization by reducing mineralization, diffusion, and mass flow of nutrients in the soil (Table 1). Drought reduces cell membrane permeability, transpiration flux, and active transport, resulting in restricted nutrient mobilization throughout the plant [6]. Specifically, water deficit hinders uptake by decreasing phosphorus distribution to roots, while also causing sugar accumulation and a decrease in leaf nitrogen content, leading to a C/N imbalance [41,42,43].

#### 2.2.2. The Biochemical Effects of Drought on Wheat

Drought stress severely disrupts wheat biochemistry by inducing reactive oxygen species (ROS) accumulation, degrading photosynthetic pigments, and forcing a metabolic shift toward osmoprotectant accumulation [44]. These biochemical alterations damage cellular membranes and proteins, though wheat activates complex antioxidant and osmotic adjustment defenses to mitigate the stress [45]. Water deprivation disrupts the equilibrium of ROS production inside plant cells (Table 2), leading to an excessive accumulation of hydrogen peroxide (H_2_O_2_) and subsequent oxidative stress [46]. This ROS accumulation diffuses across cell biomembranes and causes oxidative damage to vital macromolecules, ultimately leading to lipid peroxidation and programmed cell death [47,48,49]. The extent of this cellular damage is biochemically tracked by measuring malondialdehyde (MDA) content and electrolyte leakage, both of which increase significantly under drought conditions [50,51]. In sensitive wheat genotypes, the antioxidant system is not properly activated, resulting in heightened MDA production and accelerated drought-induced leaf senescence [52,53]. Progressive drought severity differentially elevates these stress markers, with MDA and H_2_O_2_ levels increasing alongside the duration of water withdrawal (Table 2). This severe cellular damage is further evidenced by a significant decline in the membrane stability index (MSI) across exposed wheat genotypes [50,54]. Drought stress forces a biochemical shift toward osmotic adjustment, primarily through the accumulation of compatible solutes like proline and soluble sugars [55]. Proline accumulation increases substantially under drought, particularly during sensitive stages like anthesis, helping plants regulate osmotic balance and defend against oxidative stress [56]. Exposing wheat plants to progressive drought also results in higher soluble sugar accumulation, which serves as an energy reserve and osmoprotectant [50]. Concurrently, drought severely degrades photosynthetic pigments, causing a significant decrease in chlorophyll a, chlorophyll b, and total carotenoid contents [57]. This pigment degradation directly reduces the photosynthetic rate and carboxylation efficiency by perturbing the photosystems and limiting carbon assimilation [45]. Tolerant genotypes counteract this by maintaining slower chlorophyll degradation and higher carotenoid levels, which protect against photooxidative damage [51,54].

#### 2.2.3. The Morphological Effects of Drought on Wheat

**Figure 1:**
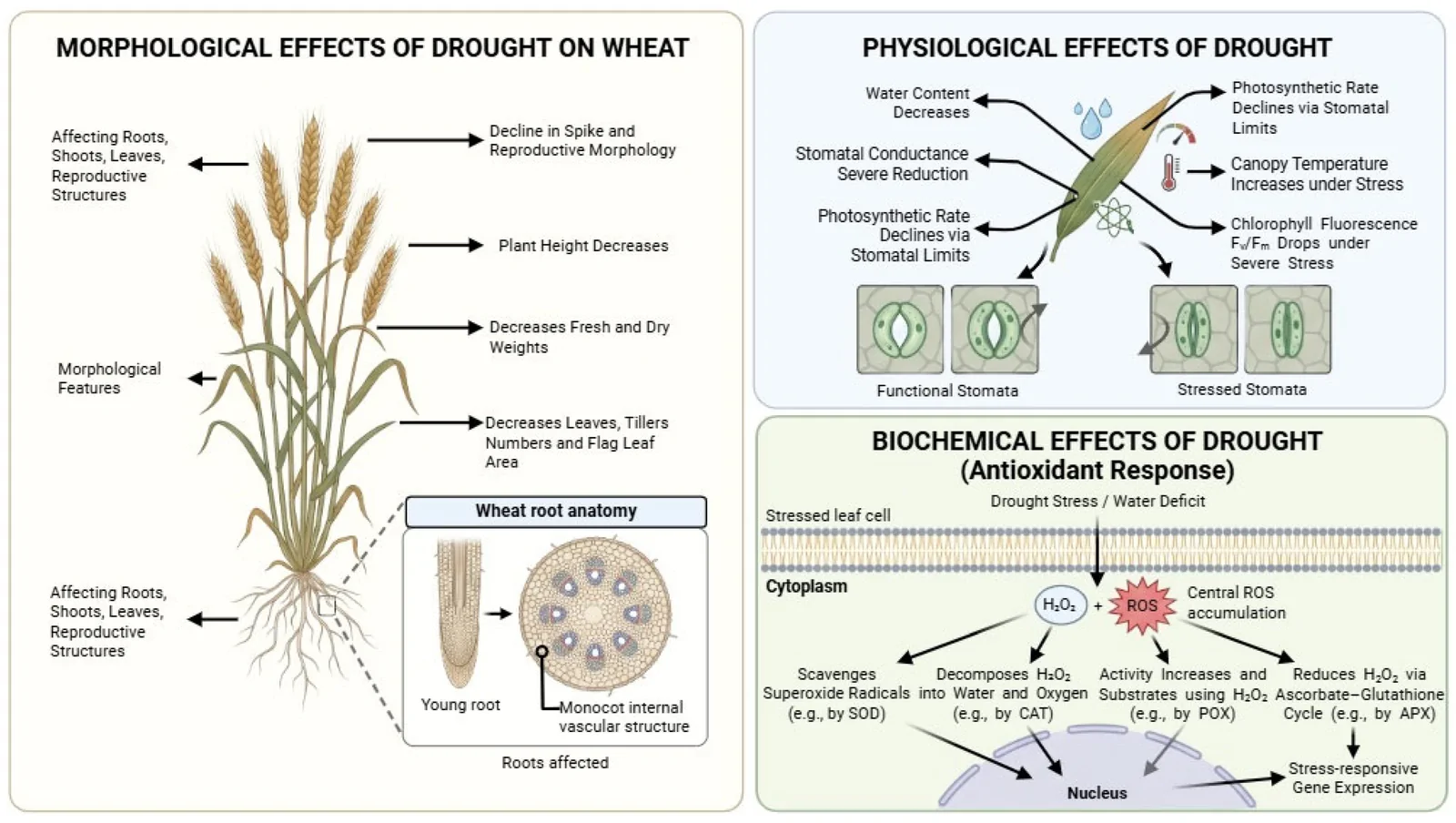
Drought effects on wheat morphology, physiology and biochemistry. In “Morphological effects” (**Left** Panel) the arrows represent Anatomical Point which is linked to specific text description directly affected plant organ, e.g., pointing to spike, stem, leaves, and roots. In “Physiological effects” (**Top Right** Panel) the arrows represent Indicator Points (Canopy Temperature increases and water content decreases) and State Transmission (Healthy stomata to Stressed Stomata). In Biochemical effects (**Bottom Right** Panel) top arrows represent stress induction (Initial impact of drought stress penetrating into cytoplasm), Downstream pathways (The four diverging arrows out from the central circle represent the activation of distinct antioxidant pathways triggered by the accumulation of reactive oxygen species) and the final downward arrow represent, end result of the Ascorbate-Glutathione pathway.

Drought stress imposes widespread morphological constraints on wheat (*Triticum aestivum* L.), affecting roots, shoots, leaves, and reproductive structures in genotype, severity, and growth-stage-dependent ways [12]. The magnitude of morphological change depends on drought timing, duration, and intensity, with stem elongation, booting, anthesis, and grain filling representing the most vulnerable developmental windows [25]. Drought reshapes wheat root architecture, anatomy, and internal vascular structure through multiple interconnected mechanisms (Figure 1). Primary root elongation declines under osmotic stress, with the traditional cultivar *Saragolla* showing greater reduction than the modern variety Svevo [58]. Lateral root growth is more reduced than adventitious root growth under water stress, with reductions of 86% and 67%, respectively, across both drought and flooding conditions. Drought also causes aggregation or cross-linking of molecules, producing larger mass fractions alongside degradation products of lower molecular mass [59]. Aboveground morphological structures undergo significant dimensional reductions under drought stress. Plant height decreases by 35% when drought occurs during stem elongation, 23% during booting, and 7% during grain filling, demonstrating strong growth-stage dependency [25]. A meta-analysis of winter wheat confirms a 10.4% average reduction in plant height under drought [16]. Drought conditions decline plant height, fresh and dry weights, leaves and tillers numbers, and flag leaf area [60]. In Atta Habib (*Triticum aestivum* L.), a widely cultivated South Asian spring wheat variety, exposure to drought stress for 7 days results in a reduction in shoot weight to 93.1 mg compared to the same-aged control plants’ shoot weight of 113 mg and shoot length to 11.1 cm, compared to the control plants’ 12.3 cm [61]. Leaf morphology responds sensitively to water deficit. Under mild drought, leaves atrophy, curl, thin, and decrease in area [62]. A meta-analysis reports a 23.4% reduction in leaf area, substantially greater than the 10.4% height reduction [22]. Severe drought significantly reduces stem growth, producing weak plants with diminished crop yield [62]. Leaf rolling and increased trichome density and leaf waxiness serve as adaptive morphological strategies to reduce transpiration loss [63]. Drought-tolerant genotypes exhibit a stay-green phenotype that delays senescence and maintains photosynthetic capacity longer [24]. Spike and reproductive morphology decline sharply under drought stress. Spike length decreases by 28.6%, spikelet number by 15.6%, grain number by 30.3%, spike weight by 23.5%, and grain weight per spike by 37.5% [57]. Thousand-kernel weight declines by 10.6% compared to irrigated conditions, with meta-analysis confirming a 3.8% reduction [22]. Drought also reduces peduncle length, biomass, and harvest index across durum wheat genotypes [64].

### 2.3. Drought Tolerance Mechanism

#### 2.3.1. Physiological Tolerance Mechanism

**Figure 2:**
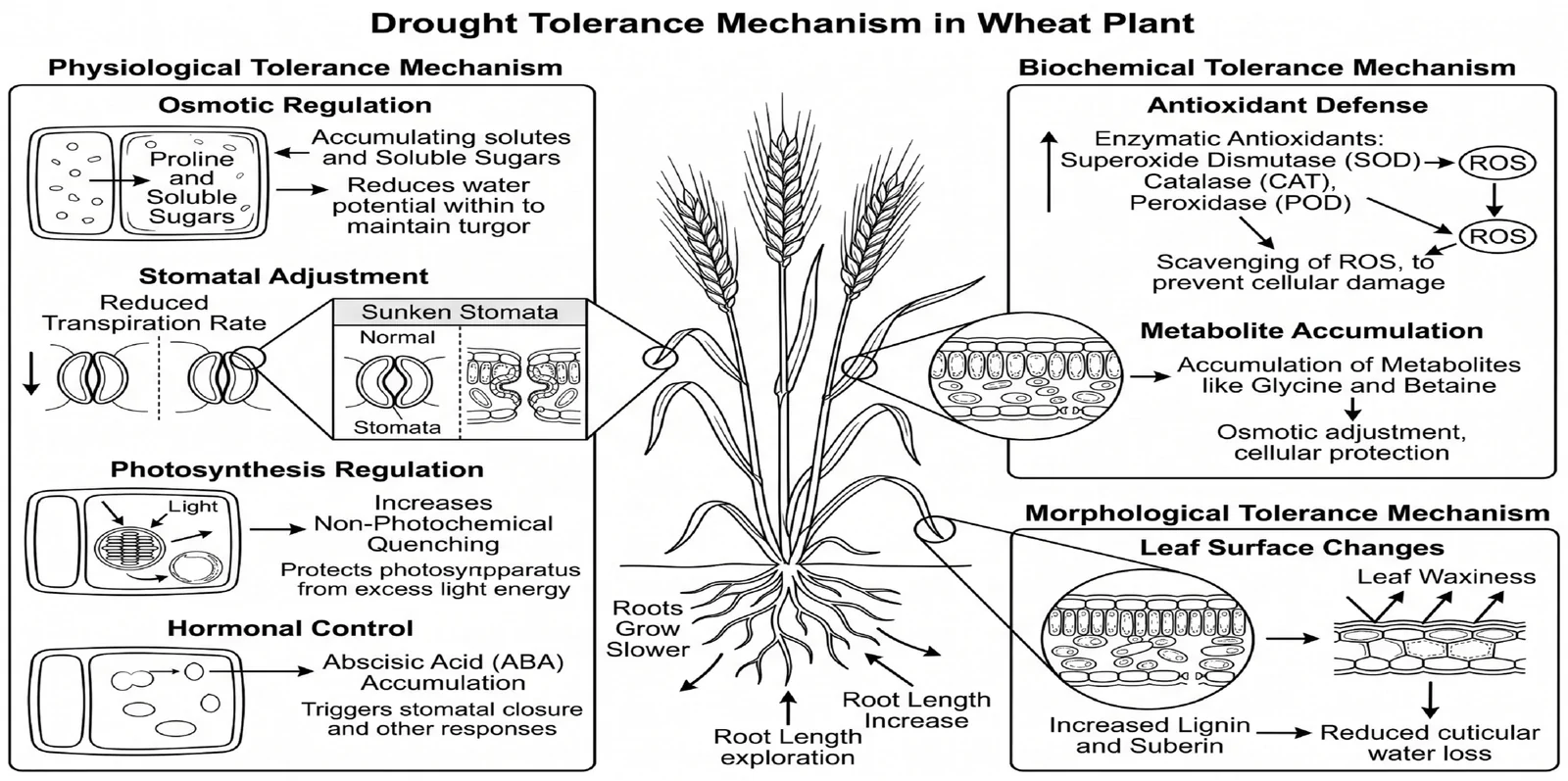
Schematic diagram illustrates the tolerance mechanism of wheat plant. In this figure the horizontal and downward arrows show “Cause and Effect Relationships”, the vertical arrows indicate quantitative increase or decrease, the anatomical arrows show physical growth and barriers (The three arrows pointing in different directions around the roots represent physical growth behavior, the roots extending downward and outward to explore deeper soil layers for water, even if overall growth rate slows. The small diagonal arrows pointing outward from the top cell layer under “Leaf Waxiness” depict the secretion and deposition of a physical wax layer on the outside of the leaf surface) and the arrows pointing from the enzymes (SOD, CAT, POD) toward ROS (Reactive Oxygen Species) show biochemical targeting and neutralization.

To cope with these water-limited environments, wheat plants deploy a battery of complex physiological defense mechanisms ranging from stomatal adjustments to osmotic regulation [63,65]. To counter this cellular dehydration, wheat plants activate osmotic adjustment (Figure 2) by accumulating compatible solutes like proline and soluble sugars, which help maintain turgor pressure and cellular function [32,34]. Leaf soluble sugar accumulation serves as the major component of this osmotic adjustment in drought-stressed wheat plants. In some genotypes, solute accumulation combined with an increase in cell wall rigidity enhances soil water uptake and limits leaf water loss [32]. Exogenous application of osmoprotectants like proline can further support this natural osmoregulation, lower leaf water potential, and support more efficient stress memory for future drought events [35]. Stomatal conductance is highly sensitive to environmental water changes, making it a critical trait for evaluating drought resistance [22]. Isohydric wheat species maintain constant leaf osmotic potential through sensitive stomatal regulation and reduced transpiration, relying more on stored carbohydrates to meet carbon demands. Conversely, anisohydric plants show marked drops in osmotic potential and reach more negative xylem water potentials as stomata close later [32]. Drought also reduces the quantum yield of electron transport, though its effects on the overall efficiency of PSII can sometimes be insignificant [22]. This imbalance between photochemical activity and Calvin cycle electron demand results in excess excitation energy and subsequent photoinhibition damage to the PSII reaction centers [42]. To dissipate this excess light energy, plants increase non-photochemical quenching, a critical photoprotection mechanism that helps maintain photosynthesis under water-deficit conditions [31,36]. Tolerant wheat varieties mitigate this damage by maintaining increased thylakoid membrane fluidity and enhancing cyclic electron transport around PSI [39]. ABA accumulation is a central hormonal response that signals drought stress and regulates stomatal closure to prevent water loss [65]. Transcriptional analyses of key ABA-responsive genes confirm that ABA signaling plays a major role in the adaptation of wheat to water deficit [10]. In resistant wheat genotypes, the expression of aquaporin genes like PIP2:1 is significantly upregulated, whereas sensitive genotypes exhibit higher expression of abscisic aldehyde oxidase (AAO) genes involved in ABA biosynthesis [32]. Wheat genotypes exhibit differential physiological resilience, making trait screening a viable strategy for drought mitigation [33,38]. Tolerant cultivars maintain higher relative water content, membrane stability, and photosynthetic pigment levels, which consequently reduces grain yield loss [34]. Wild relatives like *Aegilops tauschii* and *Ae. speltoides* demonstrate pronounced resilience by maintaining physiological integrity and enhancing antioxidative enzyme activity under water-limited conditions [38]. Physiological damage can be partially reversible; re-watering after short-term drought restores stomatal conductance and photosynthesis to near control levels. However, prolonged drought inflicts lasting damage on photosystems I and II, preventing full photosynthetic recovery. The recovery ability of field-grown wheat is generally better than that of pot-grown wheat, and recovery is inversely proportional to stress intensity [15,22]. Silicon application can improve root systems and positively impact canopy physiology, significantly enhancing photosynthesis, stomatal conductance, and transpiration under drought [37]. Ultimately, understanding these complex physiological interactions is essential for developing drought-resistant crop varieties and ensuring agricultural sustainability [36].

#### 2.3.2. Biochemical Tolerance Mechanism

To combat ROS-induced oxidative damage, wheat plants activate a robust enzymatic antioxidant defense system [45]. The primary enzymatic antioxidants superoxide dismutase (SOD), catalase (CAT), and peroxidase (POD) increase their activities significantly in response to drought stress [47,48]. Drought-tolerant wheat varieties exhibit enhanced antioxidant enzyme capacity, which successfully reduces ROS levels and diminishes membrane lipid damage compared to sensitive varieties [49,54]. The ascorbate glutathione cycle also plays a critical role in ROS detoxification, where enzymes like dehydroascorbate reductase (DHAR) and glutathione reductase (GR) reinforce the plant’s defense system under severe water deficits [51]. However, antioxidant activation is metabolically costly; drought-rust stress elevates catalase activity, but this biochemical defense is directly linked to decreased morphological growth traits [10]. Drought induces complex metabolic reprogramming in wheat, altering nitrogen assimilation, endogenous hormone levels, and specialized metabolite pathways [48,66]. ABA signaling plays a major role in drought adaptation, with transcriptional analyses confirming its central involvement in the biochemical response to water deficit. Metabolome analysis identifies specific drought-responsive biomarkers, including enhanced nitrogen recycling through purine and pyrimidine metabolism and an anti-senescence response driven by serotonin accumulation under severe stress [10]. The protein profile varies dynamically under drought, with SDS-PAGE indicating that the abundance of certain protein spots is downregulated while others are upregulated to manage cellular stress [45,60]. Drought also impacts nutrient assimilation enzymes; restricted potassium availability reduces nitrate reductase (NR) activities, worsening the biochemical stress [66]. Furthermore, drought alters the glyoxalase system, and mitigating this damage requires upregulating Glyoxalase I (Gly I) and Glyoxalase II (Gly II) enzymes to detoxify methylglyoxal, a toxic byproduct of altered metabolism [46].

#### 2.3.3. The Morphological Tolerance Mechanism

Adventitious roots grow slower and reach only one-third of control length under water stress [67]. Root dry weight decreases significantly [68]. Despite overall root biomass reduction, root length can paradoxically increase during active drought as plants prioritize root elongation for water foraging, reaching 14.0 cm under stress versus 13.4 cm during recovery [61]. Drought-tolerant genotypes increase root vessel number and root density to improve water transport and absorption [69]. The traditional *Saragolla* cultivar revamps its root architecture by significantly increasing lateral root length and root hair density compared to the modern Svevo cultivar [58]. Anatomical remodeling includes a 33% decrease in the xylem-to-stele ratio and a 2.1-fold increase in the aerenchyma-to-cortex ratio under water stress (Figure 2). Drought triggers increased lignin and suberin deposition in the endodermis, forming apoplastic barriers that reduce radial water loss [67]. Cross-section analysis reveals enlargement of the vascular cylinder and dense distribution of xylem vessels under drought [70]. Scanning electron microscopy of the tolerant *Saragolla* cultivar shows a greater stele area and increased xylem lumen vessel size under osmotic stress [58]. At the cellular level, drought induces remodeling of cell wall architecture within just five days, involving hydroxyproline-rich glycoproteins, arabinoxylan, and pectic compounds. Deposition of unesterified and esterified homogalacturonans and arabinogalactan proteins indicates active cell wall reconstruction as a drought-prevention mechanism. Higher levels of unesterified homogalacturonans permit calcium cross-linking, which enhances cell wall rigidity and aids intracellular water preservation. The wheat plant accelerates its life cycle to escape drought, reflecting an overall decrease in developmental timing [6]. Morphological drought tolerance involves an integrated suite of adaptive traits. A deep and branched root system is a fundamental component of drought tolerance, and root morphological plasticity influences whole-plant growth [58]. However, an excessively high root–shoot ratio reduces water use efficiency and is not conducive to high yields [16]. Screening morphological features at early developmental stages provides valuable insights into late growth performance linked to productivity [12]. Drought-induced morphological changes in wheat are pervasive, genotype-dependent, and span molecular to whole-plant scales (Figure 2). Root anatomical remodeling, including xylem vessel redistribution, aerenchyma formation, and endodermal suberization, represents a core adaptive response, while aboveground reductions in leaf area, plant height, and spike dimensions directly constrain yield. Breeding efforts targeting genotype-specific root architecture, stay-green phenotypes, and optimized root–shoot partitioning offer the most promising morphological pathways to drought-resilient wheat.

## 3. Discussion

### 3.1. Genetic Management of Drought Stress

#### 3.1.1. TaSnRK Genes Orchestrate Wheat Drought Tolerance

Sucrose non-fermenting 1-related protein kinases (SnRKs) act as central signaling hubs in wheat *(Triticum aestivum* L.) drought response, coordinating stomatal regulation, osmotic adjustment, and ROS scavenging [71,72,73]. Drought stress upregulates multiple SnRK family members, triggering phosphorylation cascades that enhance plant survival and yield under water-limited conditions [74,75]. TaSnRK2 family members serve as essential components of the ABA signaling pathway initiated by osmotic stress (Table 3), triggering downstream drought responses through phosphorylation cascades [74,75]. TaSnRK2.4 forms a functional TaPP2C01-TaSnRK2.4-TaABF2 module with upstream and downstream partners, where TaSnRK2.4 and TaABF2 positively regulate drought tolerance while TaPP2C01 acts negatively by modulating stomatal movement, osmotic adjustment, ROS homeostasis, and root morphology [74]. Overexpression of TaSnRK2.4 in Arabidopsis results in longer primary roots, higher yield under normal conditions, and enhanced tolerance to drought through decreased water loss rates and improved osmotic potential [76]. TaSnRK2.8-5A forms a novel ABA signaling module with TaPP2C53-1A and TabZIP23-6D, where overexpression of TaSnRK2.8-5A or TabZIP23-6D alleviates drought-induced growth inhibition while TaPP2C53-1A acts as a negative regulator [77]. TaSnRK2.1-2D enhances drought tolerance by promoting aerenchyma formation and interacting with catalase (TaCAT-1A) to modulate ROS scavenging [78]. Sense overexpression of TaSnRK2.1 enlarges plant growth phenotypes, increases dry weights, promotes stomata closing, and elevates cellular protective enzyme activities under drought treatment, while antisense expression reduces these parameters [79]. TaSnRK2.5-2D interacts with TaPP2C62-2A and bZIP transcription factor TaABI5-3B to establish a regulatory module where TaSnRK2.5-2D and TaABI5-3B positively regulate plant growth and agronomic traits under drought conditions [80]. TaSnRK2.8 overexpression in Arabidopsis confers enhanced tolerance to drought, salt, and cold stresses, supported by longer primary roots, higher relative water content, strengthened cell membrane stability, and significantly lower osmotic potential [81]. TaSnRK3.23B, a calcineurin B-like protein kinase (CBL), promotes ROS scavenging via accumulation of antioxidant enzymes, including SOD, CAT, and POD, conferring significant tolerance to drought. TaSnRK3.23B is detected on the cell membrane and in the nucleus and interacts with calcineurin B-like proteins TaCBL2B and TaCBL6B [82]. TaSnRK genes offer valuable tools for wheat breeding through haplotype-specific markers and association analysis. The specific haplotype TaSnRK2.8-5A-Hap1 confers superior drought tolerance, and TaSnRK2.8-5A overexpression lines display plant biomass increases of 32–42% compared with wild-type plants under drought treatment [75]. TaSnRK2.9 gene expression studies reveal involvement in response to PEG, NaCl, low temperature, and ABA treatments, confirming its multi-stress responsiveness [73]. TaSnRK2.8-A gene alleles show significant differences between wheat varieties in yield under drought and irrigation conditions (Table 3), with varieties carrying the G allele having higher yields under irrigation and drought conditions in 2018. The Koshova variety was the most productive under drought conditions and remained one of the most productive under irrigation. Yields in drought and drought resistance index depend on the interaction of genotype and environmental factors [83]. Transcriptomic analysis identifies SnRK2 as a key regulator in drought response, with tolerant cultivars demonstrating robust antioxidant defense and stable CAT activity mitigating oxidative damage [84]. The TaSnRK gene management of drought tolerance in wheat operates through coordinated phosphorylation cascades, ABA-dependent signaling modules, and ROS scavenging networks. The diversity of TaSnRK family members from TaSnRK1α controlling root architecture to TaSnRK2.10 regulating stomatal aperture and phosphoenolpyruvate (PEP) supply provides multiple targets for engineering drought-resilient wheat cultivars. Superior haplotypes identified across TaSnRK genes offer practical molecular markers for breeding programs aimed at enhancing wheat productivity under increasingly arid conditions.

#### 3.1.2. TabZIP156 Enhances Wheat Drought Tolerance

Among the various transcription factors involved in the wheat drought response, the specific functions of many basic leucine zipper (bZIP) transcription factors related to drought tolerance are still not well understood [85,86]. The bZIP transcription factor TabZIP156 acts as a positive regulator in response to drought tolerance in Arabidopsis and wheat, offering valuable insights for breeding drought-resistant cultivars (Table 4). TabZIP156 is highly expressed in both roots and leaves, and it responds significantly to drought and ABA stress (Table 4). Through subcellular localization and transactivation assays, overexpression of TabZIP156 in Arabidopsis enhanced drought tolerance, as evidenced by a higher germination rate, longer root length, lower water loss rate, and reduced ion leakage [87]. These physiological modifications align with broader drought response mechanisms in wheat, where protein phosphorylation, oxidation reduction, and regulation of transcription are top biological processes involved in drought response and tolerance [88]. ROS homeostasis is maintained by TabZIP156 through upregulation of antioxidant enzymes, including POD, SOD, and CAT [87]. Drought tolerance in wheat is a complex trait regulated by multiple genes, requiring integrated approaches to identify important gene modules and metabolite pathways [88,89,90]. Stress tolerance is polygenic, governed by numerous genes that exert minor effects and exhibit significant genotype–environment interactions, making understanding drought tolerance at the molecular level very complex [88].

#### 3.1.3. TaNAC6-3B Drives Wheat Drought Tolerance

Functional characterization of the candidate NAC family transcription factor TaNAC6-3B revealed its nuclear localization, positioning it directly within the cellular compartment where transcriptional regulation occurs (Table 5). This nuclear targeting aligns with the broader family of plant-specific NAC transcription factors, which have been reported to play roles in diverse stress responses and developmental processes [91]. Mechanism studies revealed that TaNAC6-3B activates the expression of the LEA (late embryogenesis abundant) protein gene TaLEA1-2B via direct binding to its promoter [92]. Transcriptome profiling of transgenic overexpression lines showed upregulation of NCED and ABA-responsive genes alongside other drought-responsive genes [93]. TaNAC6-3B overexpression significantly enhanced drought tolerance in transgenic wheat lines [92]. TaNAC071-A knockdown in wheat attenuated plant drought tolerance, whereas its overexpression enhanced it [91].

#### 3.1.4. TaLBD16 Positively Regulates Wheat Drought Tolerance

Transcription factors act as key regulatory switches controlling gene expression across diverse biological and metabolic processes [94]. Among these regulators, lateral organ boundaries domain (LBD) proteins function as plant-specific transcription factors that play vital roles in regulating plant growth, development, and tolerance to abiotic and biotic stress. The detailed molecular mechanism of LBD proteins in adjusting wheat drought resistance remained unclear until recent characterizations of TaLBD16. TaLBD16 is strongly induced by both drought and ABA treatments, situating it directly within the core hormonal stress response pathway [71]. This functional activation aligns with broader transcription factor regulatory networks, where a gene encoding a specific TF and its target genes constitute a regulon that participates in signal transduction to activate drought response pathways [95]. Understanding the role and regulation of such transcription factors facilitates crop improvement strategies aimed at developing agronomically superior, drought-tolerant plants [94].

#### 3.1.5. TaDREB26-B Enhances Wheat Drought Tolerance

Dehydration-responsive element-binding (DREB) transcription factors, members of the AP2/ERF superfamily, are pivotal regulators of stress-responsive gene expression by binding to DRE/CRT cis-elements [77]. These transcription factors represent promising targets for engineering crop resilience, as they control the activity of multiple stress response genes in a coordinated manner [96]. DREB TFs play a crucial role in enhancing abiotic stress tolerance in plants by regulating the expression of stress-inducible genes through interaction with these cis-elements [97]. Among these regulators, TaDREB26-B has emerged as a prime regulator for improving drought tolerance in wheat. TaDREB26-B expression was rapidly upregulated by both drought and ABA treatments, situating it directly within the core hormonal stress response pathway [98]. The role of DREB1 in drought resilience is further supported by protein–protein interactions with peroxidase, highlighting a range of protein sizes and stability indices across TaDREB proteins in the wheat genome [99].

#### 3.1.6. The TaMYB2-TaMAP3K17 Signaling Module and TaPHL7-TaSOD7-4A Antioxidant Regulatory Gene Pairs Drive Wheat Drought Tolerance

The mitogen-activated protein kinase (MAP3K) TaMAP3K17 was identified as a drought-induced gene in wheat, encoding a protein localized to both the plasma membrane and nucleus [100,101,102]. Virus-induced gene silencing (VIGS) of TaMAP3K17 generated wheat plants that were hypersensitive to drought stress and accumulated higher levels of ROS compared to the wild type [100]. Although SOD proteins are known to enhance drought resistance, the upstream regulatory mechanisms governing their expression remained poorly understood until the characterization of the TaPHL7-TaSOD7-4A pair [103]. Wheat employs a diverse array of transcription factor and target gene pairs to manage drought tolerance beyond the TaMYB2-TaMAP3K17 and TaPHL7-TaSOD7-4A modules. The AP2/ERF transcription factor TaRAP2-13L interacts with TaWRKY10 to synergistically enhance drought tolerance by activating the expression of TaSOD3-2A, TaSOD3-2D, TaGPX1-D, and TaNCED2-5B [104]. The TaMYB2-TaMAP3K17 and TaPHL7-TaSOD7-4A modules operate within a broader network of ABA signaling and kinase pathways that coordinate wheat drought responses [105]. The physiological outcomes of these gene pair modules converge on several key drought tolerance mechanisms, including ROS scavenging, osmotic adjustment, and water retention. In the TaMYB2-TaMAP3K17 module, antioxidant enzyme activities (POD, CAT, and APX) were significantly higher in overexpression lines than in wild-type plants [100]. Similarly, the TaPHL7-TaSOD7-4A pair enhances antioxidant enzyme activity while reducing H_2_O_2_ and MDA contents [103].

### 3.2. Treatments

#### 3.2.1. Abscisic and Jasmonic Acids Mitigate Wheat Drought Stress

To cope with water deficit, plants employ phytohormones like ABA and jasmonic acid (JA) to redesign physiological and biochemical signal transduction cascades [106,107,108]. These chemical acids manage drought tolerance by modulating stomatal closure, activating antioxidant enzymes, and regulating osmolyte accumulation [109]. ABA is a premier signal for plants to respond to drought, triggering stomatal closure to reduce water loss by evapotranspiration [110,111]. Exogenous ABA application by root irrigation increases leaf ABA accumulation, which decreases leaf stomatal conductance and triggers early stomatal closure in winter wheat [112]. ABA accumulation in the leaf apoplast induces stomatal closure, preventing intracellular water loss, though this also decreases carbon assimilation and impairs photosynthesis [110]. Under terminal drought, the plant hormone ABA is considered the major chemical signal involved in stomatal regulation, and wheat genotypes differ in their ability to produce ABA and in stomatal sensitivity to ABA [111]. ABA triggers many physiological processes, including bud dormancy, seed germination, and transcriptional and post-transcriptional regulation of stress-responsive gene expression [107]. Furthermore, ABA helps seeds overcome stress conditions and germinate only when conditions are suitable [110]. Exogenous ABA application significantly improves wheat growth and yield attributes under drought stress [112,113]. Foliar application of ABA and glycine betaine under drought considerably increased the rate of photosynthesis and pigment contents in flag leaves compared to plants grown under individual drought conditions. ABA and glycine betaine application significantly enhanced catalase and peroxidase enzyme activities and total antioxidant capacity, reducing oxidative damage and increasing grain yield [114]. Seed and root priming with ABA and glycine betaine also enhanced drought tolerance in wheat seedlings by improving seedling growth and antioxidative defense. Under drought, root and shoot length and biomass were significantly increased in ABA-primed seedlings compared to non-primed seedlings. ABA-primed seedlings showed reduced lipid peroxidation and proline content, suggesting a declined state of oxidative damage [115]. Jasmonic acid is an endogenous growth-regulating substance initially identified as a stress-related hormone in higher plants. The concentrations of endogenous JAs increase rapidly following drought stress and then return to baseline levels if stress periods are prolonged. Jasmonic acid can minimize water loss by regulating stomatal opening and closing [109]. Application of exogenous jasmonates significantly alleviates drought damage in wheat by markedly reducing malondialdehyde content, O_2_^−^ and H_2_O_2_ accumulation. Exogenous jasmonates reduce both osmotic regulators and MDA content, suggesting alleviated plant damage under drought conditions. Activation of JA biosynthesis and JA-mediated signaling pathways acts as a key factor for enhancing drought resistance in wheat [116]. Jasmonic acid might regulate the response to drought stress in wheat by controlling secondary metabolism, including phenylpropanoid biosynthesis and terpenoid biosynthesis. Transcriptome analysis reveals a high dynamic response to jasmonates and drought conditions in wheat seedlings, especially in root tissue. Diterpenoid biosynthesis shows a high relationship with gibberellin and abscisic acid biosynthesis, potentially contributing to drought resistance [116]. JA application improves chlorophyll and relative water content under PEG-induced drought stress. JA performance was found to be better than ABA under PEG-induced drought stress in pearl millet [117]. Drought priming upregulates genes encoding the biosynthesis and metabolism of ABA and JA, as well as genes involved in their signaling pathways. Endogenous concentrations of both JA and ABA increase following drought priming [108].

#### 3.2.2. Nanoparticles Mitigate Wheat Drought Stress via Antioxidant Activation

Nanoparticles (NPs) have emerged as an excellent tool to enhance crop production under rapid climate change and increasing drought intensity. Drought stress negatively affects plant growth, disturbs cellular membranes, and impairs nutrient and water uptake, photosynthetic apparatus, and antioxidant activities. The application of NPs protects membranes, maintains water relationships, and enhances nutrient and water uptake, leading to an appreciable increase in plant growth under drought conditions. NPs protect the photosynthetic apparatus and improve photosynthetic efficiency, accumulation of osmolytes, hormones, and phenolics, antioxidant activities, and gene expression, providing better resistance to plants against drought stress [118]. A meta-analysis of 83 peer-reviewed investigations demonstrated that NP applications under drought stress significantly enhanced crop growth and improved water use efficiency by 28.3% and 52.4%, respectively. Among various NP types, ZnO NPs demonstrated the highest efficacy in enhancing drought resilience across crop types [119].

a. Silicon and Silica Nanoparticles

Silicon nanoparticles (Si-NPs) have shown their potential for use in farming under water-deficient conditions. The Si-NPs improved leaf gas trade attributes and chlorophyll a and b concentrations while decreasing oxidative pressure in leaves, demonstrated by diminished electrolyte leakage and enhanced SOD and peroxidase activities [120]. SiO_2_ nanopriming enhanced the ability of wheat plants to withstand water-deficit conditions by balancing the production of ROS and the activity of enzymatic antioxidants like peroxidase, catalase, and SOD. Nanoprimed plants had a higher number of active reaction centers, high absorbance, trapping, and electron transport rates compared with unprimed plants under drought conditions [121].

b. Zinc Oxide Nanoparticles

ZnO-NP application effectively mitigated the harmful effects of drought by enhancing both root and shoot lengths. ZnO-NPs emerged as a potential drought alleviator and yield-oriented safe nano-fertilizer for wheat in semi-arid regions facing irrigation challenges [122]. Soluble protein content increased in response to nanoparticles under drought stress but not in well-watered conditions, and the application of nanoparticles at 40% field capacity induced a stress-resistant protein band [123]. ZnO nanoparticle-based nanopriming improved germination and seedling establishment under both NP and NP plus drought conditions [124].

c. Selenium Nanoparticles

SeNPs improved the photosynthetic pigments, photosynthetic rate, gas exchange, and transpiration rate of wheat plants and enhanced drought tolerance by increasing the activity of antioxidant enzymes, including catalase (CAT), ascorbate peroxidase (APX), and SOD, and the expression level of stress-responsive genes [125]. Foliar spraying with Se(NPs) and Se significantly improved tissue water retention and the fresh weights after Se(NPs) treatment compared to untreated controls. The application of Se(NPs) was notably more effective than the application of Se in mitigating drought stress, indicating the potential of the application of Se(NPs) as a sustainable agricultural practice under water-limited conditions [126]. Selenium nanoparticles at low concentrations were found to have prominent bioactivity and biosafety properties and were known to be nontoxic and biocompatible compared to their counterparts, selenate and selenite [125].

d. Silver and Copper Nanoparticles

AgNPs treatment leads to a controlled ROS accumulation, which activates the antioxidant system, reduces lipid peroxidation, and enhances photosynthetic efficiency under drought conditions. AgNPs application significantly increases grain yield, with accumulation primarily in leaves and stems and no detectable presence in grains, suggesting a low risk of contamination in the food chain. Spraying AgNPs produces ROS, which triggers the wheat stress response and forms stress memory, enabling wheat plants to respond rapidly to subsequent drought stress [127]. CuNPs and AgNPs treatment resulted in significantly higher chlorophyll stability index, leaf succulence, and leaf K content in plants, indicating their positive role in drought tolerance of wheat [128].

e. Composite and Iron Nanoparticles

Zinc Cobalt Sulfide (ZCS) nanoparticles have the ability to improve the osmotic status of plants, enhance the synthesis of osmoprotectants, activate ROS scavenging enzymes for maintaining membrane integrity and cellular protection, and promote yield increment during drought stress [129]. Iron nanoparticles (Fe-NPs) improved the photosynthesis, yield, and Fe concentrations and diminished the Cd concentrations in tissues of wheat under combined cadmium and drought stress. Fe-NPs alleviated the oxidative stress in leaves, and the efficiency depends on the NPs’ concentrations applied in the soil [130]. The presence of NPs inside the plant tissues can restrain oxidative stress enzymes and trigger the plant defense system. NPs influence the plants by a two-phase dose-reaction “hormesis” with an up-dose inhibition and low-dose stimulation [122]. NPs not only enhance the production of antioxidants but also increase the relative water content in stressed plants, reduce biomass loss, and improve the drought resistance index. The application of chitosan nanoparticles enhances biochemical profiling and modulates the genes involved in the drought tolerance of plants [19].

#### 3.2.3. Potassium Application Mitigates Wheat Drought Stress

Potassium (K) application is known to alleviate drought stress in crops [77,131,132]. Plants suffering from drought stress require more internal potassium, and potassium plays a vital role in improving the plant resistance [133,134]. Low K in plant tissues is known to aggravate the effects of drought stress by impairing the osmoregulation process and the photosynthetic carbon metabolism [135]. Water deficit may increase cellular membrane permeability, resulting in K outflow, and internal K starvation may disorder plant metabolism and limit plant growth [136]. The beneficial effect of K on cereal growth under drought has been related to maintenance of water balance in the crops, increased activity of the antioxidant enzymes, increased synthesis of osmolytes, contribution to a low sodium (Na)/K ratio, extension of the grain filling period, and increase in the grain growth rate and weight [132]. Potassium enhances drought tolerance in plants through mitigating harmful effects on translocation, osmoregulation, photosynthesis, transpiration, maintaining water balance, stomatal opening and closing, and synthesis of protein [133]. Potassium plays an essential role in osmotic regulation, photosynthesis, and nitrogen assimilation, all of which are critical for maintaining plant growth and productivity under stress conditions [66]. The yield-limiting effect of water deficit could be overcome by increasing K supply [133,134]. K supply could be exploited as a tool for increasing cereal tolerance to drought, for example, by considering late administration of K through top dressing applications or foliar sprayings [132]. K operates by enhancing various mechanisms, encompassing osmotic adjustment, hormone regulation, nutrient uptake, maintenance of photosynthetic rate, and overall stress tolerance [137]. The exogenous application of K under drought stress at all three critical growth stages enhanced tolerance of wheat by reducing toxic nutrients’ uptake and improving the physiological efficiency [134]. Drought priming and K application improved wheat performance by enhancing osmotic adjustment (OA) and leaf hydraulic conductance (KL), respectively, although the underlying mechanisms differed. The combination of drought priming and K application synergistically promoted OA and KL, thereby minimizing leaf water loss and reducing stomatal limitations on photosynthesis, which maximized grain yield under drought conditions [138]. Sufficient potassium (SK)-treated plants exhibited higher biomass, chlorophyll content, maximum quantum efficiency of photosystem II, and lower canopy temperatures, even under drought conditions [66]. Optimal K nutrition may promote partitioning of carbohydrates in stem tissues and subsequent mobilization of these carbohydrates into developing grain under drought stress. Plants with a high capacity to store large amounts of soluble carbohydrates in stems before anthesis and mobilize them into grain post-anthesis have a high potential to yield well in dry and hot environments. Because K is the mineral nutrient deposited mainly in stems, a special consideration should be given to stems of crop plants in research dealing with the effects of K on yield formation and stress mitigation [135].

### 3.3. Drought Stress Management in Wheat Through Microbial Endophytes

Endophytic fungi and bacteria colonize plant tissues and roots, enhancing drought resistance through phytohormone production, antioxidant activation, improved nutrient uptake, and stress-responsive gene induction [139].

#### 3.3.1. Bacillus Species as Drought-Protective Agents

Endophytic bacteria from the genus Bacillus represent a prominent strategy for enhancing wheat drought tolerance through multiple physiological mechanisms [140]. Bacillus species establish colonies in the root system through phytohormone production, modification of root architecture, and activation of stress-inducible genes, collectively promoting plant growth and stress resistance [141]. In durum wheat, Bacillus amyloliquefaciens and Bacillus licheniformis exhibited significant growth promotion under both normal and drought stress conditions, with isolates demonstrating high antagonistic activity and very high drought tolerance [142]. Bacterial endophytes reduce disease severity under drought, with Bacillus treatments lowering Fusarium crown and root rot severity by up to 82% under normal conditions and up to 61% under drought stress [142]. Drought-resistant wheat varieties harbor more diverse bacterial taxa, including Bacillus and Streptomyces, which function as potential keystone taxa in heightening drought resistance [143].

#### 3.3.2. Dark Septate Endophytes in Wheat Drought Tolerance

Dark septate endophytes (DSEs) are root-associated fungi that frequently inhabit nutrient-limited or high-stress environments and form symbiotic relationships promoting plant growth and modulating drought tolerance [144]. Under drought conditions, desert DSEs significantly increased grain weight, root length, root surface area, and aboveground nitrogen content of wheat host plants. Under mild drought, alternata, Paraphoma pye and Paraphoma radicina increased leaf number and plant height, while *P. radicina* showed significant beneficial influence on total biomass across all treatments [145].

#### 3.3.3. DSE Species Performance Under Drought

Microbial inoculant technology using DSEs has emerged as a promising strategy to enhance crop drought resistance and achieve high yield (Table 6). Two dominant DSE strains, *Alternaria alstroemeriae* and *Paraphoma chrysanthemicola*, significantly increased tiller number, biomass, spike length, spike weight, grain number, thousand grain weight, and grain protein and sugar content in wheat under drought [146]. Aspergillus fumigatus biopriming of wheat grains increased total biomass of stressed plants by more than 40% compared to non-bioprimed controls, alongside increased photosynthetic pigments and modulation of enzymatic and non-enzymatic antioxidants [147]. Aspergillus and Fusarium were not only abundant in root systems but also demonstrated. Phosphate-solubilizing capabilities, suggesting dual nutritional and stress-adaptive functions. Wheat roots formed a drought-specific endophytic fungal community centered around Stachybotrys, Fusarium, and Aspergillus, indirectly enhancing crop drought tolerance through community-level functional shifts [148,149]. The most abundant seed-associated fungi across cultivars, treatments, and generations included *Cladosporium, Penicillium*, Alternaria, and Epicoccum, indicating a stable core mycobiome maintained through multigenerational drought [150]. DSEs stimulate antioxidant enzyme activity including SOD and peroxidase while enhancing accumulation of osmoprotectants such as soluble sugars and promoting extracellular polymeric substance synthesis [144]. *Alternaria alternata* LQ1230 inoculation regulated antioxidant enzyme activities and compatible solute content (soluble sugars and proline) in wheat under water deficit [151]. DSE strains *Neocamarosporium phragmitis* and *Alternaria tellustris* positively influenced SOD activity and increased glutathione content under combined drought and cadmium stress [152]. DSE colonization results in increased root and shoot biomass, enhanced branching, and more vigorous leaf development, directly improving water acquisition capacity [144]. For the host plant *Hedysarum scoparium*, DSEs improved root biomass and length depending on species, with *Paraphoma* sp. and *Cladosporium oxysporum* exhibiting positive effects under all drought treatments [153]. Successful colonization of *Mortierella alpina* on root surfaces enhanced wheat drought resistance through activation of drought-responsive genes including CIPK9 and PP2C30 [143,152].

## 4. Conclusions

One of the most significant abiotic constraints limiting wheat production globally is drought stress. It causes severe and multifaceted reductions in wheat growth due to interconnected changes at various levels ranging from morphological to molecular disruption. The extent of crop yield reduction caused by drought varies depending on its intensity, duration, time point of onset relative to the plant’s growth phase and specific genotype. Continuous and terminal droughts occurring specifically during critical stages of flowering and grain-filling are usually the most devastating ones. Positive responses of crops post-drought rewatering can alleviate many of the detrimental consequences associated with water deficit conditions. Therefore, the ability to recover from drought is directly proportional to the rewatering period length but inversely related to drought severity. Because drought tolerance traits are highly polygenic and environmentally sensitive, there has been limited success using conventional breeding methods to improve wheat’s response to drought situations. Hence, combining various molecular tools like QTL (quantitative trait loci) mapping, genome editing, transcriptomic approaches alongside physiological trait-based selections would certainly contribute toward developing drought-tolerant wheat varieties. Recent studies show that biostimulants and fungal inoculants could also be effective complimentary approaches to enhance the plant’s drought stress tolerance capabilities. Integrating genetic, physio-biochemical and agronomic strategies guided by various genotype-specific tolerance mechanisms will allow future researchers to produce wheat cultivars with robust and durable resistance against drought stresses. This would ensure sustenance of global food security amidst ongoing climate change processes.

## Figures and Tables

**Table 1:** Key physiological parameters in wheat affected by drought stress.

Physiological Parameter	Drought Effect	Mechanism/Genotype Response
Relative Water Content	Significant decrease [6,32]	Tolerant genotypes maintain higher RWC via osmotic adjustment [33,34]
Stomatal Conductance	Severe reduction [29,32]	Sensitive genotypes utilize strict stomatal control to avoid turgor loss [32]
Photosynthetic Rate	Declines via stomatal limits [35,36]	Linked to lower Rubisco activity and impaired PSII function [29]
Canopy Temperature	Increases under stress [10,37]	Silicon application can reduce canopy temperature and enhance depression [37]
Chlorophyll Fluorescence	Fv/Fm drops under severe stress [38]	Tolerant varieties show lower PSII inhibition and better thermal dissipation [39]

**Table 2:** Key antioxidant enzymes and their responses to drought in wheat.

Antioxidant Enzyme	Biochemical Role Under Drought	Genotype Response
SOD	Scavenges superoxide radicals [48]	Higher in tolerant varieties [49]
CAT	Decomposes H_2_O_2_ into water and oxygen [50]	Declines in sensitive genotypes under severe stress [51]
POD	Oxidizes substrates using H_2_O_2_ [52]	Activity increases progressively with drought duration [47]
APX	Reduces H_2_O_2_ via the ascorbate glutathione cycle [51]	Upregulated alongside stress-response genes [50]

**Table 3:** TaSnRK gene types and its functions.

TaSnRK2 Gene	Downstream Partners	Physiological Mechanism
TaSnRK2.10	TaERD15, TaENO1	PEP supply, proteasomal degradation [72]
TaSnRK2.4	TaPP2C01, TaABF2	Stomatal movement, root morphology [74]
TaSnRK2.8-5A	TaPP2C53-1A, TabZIP23-6D	Proline biosynthesis, ROS scavenging [75]
TaSnRK2.1	TaCAT-1A	Aerenchyma formation, ROS modulation [78]
TaSnRK2.5-2D	TaPP2C62-2A, TaABI5-3B	Osmolyte biosynthesis, leaf water retention [80]

**Table 4:** Describes TabZIP gene’s locations and response mechanism [87].

Gene/Protein	Subcellular Location	Drought Response Mechanism
TabZIP156	Nucleus	Transcriptional activator; binds target promoters
TaJAZ3-2A	Nucleus	Antagonizes TabZIP156; negative regulator
TaP5CS	Target gene	Proline biosynthesis; osmotic adjustment
TaDREB1A	Target gene	DREB pathway activation; stress response
TaPOD	Target gene	ROS scavenging

**Table 5:** Regulatory role of TaNAC6 genes and its mechanism of action for drought stress [91,92].

Gene/Element	Regulatory Role	Mechanism of Action
TaNAC6-3B	Positive regulator	Nuclear localization; activates downstream stress genes
TaLEA1-2B	Downstream target	Late embryogenesis abundant protein; promoter bound by TaNAC6-3B
TaNAC071-A	Positive regulator	108-base pair insertion alters expression; improves water-use efficiency
TaMYBL1	Upstream activator	Binds MYB cis-regulatory elements in TaNAC071-A insertion
TaNAC2a	Positive regulator	Overexpression increases fresh and dry weight under drought

**Table 6:** Comparative drought tolerance benefits of key dark septate endophyte species inoculated in wheat, summarizing growth and yield parameters improved under drought conditions.

DSE Species	Key Drought Benefits	Wheat Parameters Improved
*Alternaria alternata*	Enhanced growth in arid areas [148]	Grain weight, root length, N content [148]
*Alternaria sorghi*	Stronger beneficial effect on growth [148]	Drought resistance, grain weight [148]
*Paraphoma radicina*	Significant biomass increases under all treatments [145]	Leaf number, plant height, total biomass [145]
*Alternaria alstroemeriae*	Increased tiller number and biomass [146]	Spike length, grain number, thousand grain weight [146]
*Paraphoma chrysanthemicola*	Yield and quality improvement [146]	Alcohol-soluble protein, soluble sugar content [146]

## Data Availability

No new data were created or analyzed in this study. Data sharing is not applicable to this article.

## Abbreviations

The following abbreviations are used in this manuscript:

ROS	Reactive Oxygen Species
MDA	Measuring Malondialdehyde
MSI	Membrane Stability Index
APX	Ascorbate Peroxidase
ABA	Abscisic Acid
SOD	Superoxide Dismutase
CAT	Catalase
POD	Peroxidase
DHAR	Dehydroascorbate Reductase
GR	Glutathione Reductase
LEA	Late Embryogenesis Abundant
LBD	Lateral Organ Boundaries Domain
NAC	A master family of plant-specific transcription factors (proteins that flip other survival genes)
JA	Jasmonic Acid
NPs	Nanoparticles
Si-NPs	Silicon Nanoparticles
SiO_2_	Silicon Dioxide
SeNPs	Selenium Nanoparticles
Se	Selenium
AgNPs	Silver Nanoparticles
CuNPs	Copper Nanoparticles
K	Potassium
Fe-NPs	Iron Nanoparticles
Cd	Cadmium
OA	Osmotic Adjustment
KL	Leaf Hydraulic Conductance
DSEs	Dark Septate Endophytes
ZnO-NPs	Zinc Oxide Nanoparticles
ZCS NPs	Zinc Cobalt Sulfide Nanoparticles
Fv/Fm	The maximum photochemical quantum efficiency of PSII
C/N	Carbon-to-Nitrogen
PSII	Photosystem II
PSI	Photosystem I
PIP2:1	Plasma membrane intrinsic protein 2:1
SDS-PAGE	Sodium Dodecyl Sulfate–Polyacrylamide Gel Electrophoresis
Gly I	Glyoxalase I
Gly II	Glyoxalase II
CBL	Calcineurin B-Like Protein
DRE/CRT	Dehydration-Responsive Element/C-Repeat
DREB	Dehydration-Responsive Element-Binding Proteins
DREB1	Dehydration-Responsive Element-Binding Proteins 1
AP2/ERF	APETALA2/Ethylene Response Factor
LQ1230	*Alternaria alternata* Strain
bZIP	Basic Leucine Zipper Protein
